# Molecular evidence of *Rickettsia* spp. in ixodid ticks and rodents in suburban, natural and rural habitats in Slovakia

**DOI:** 10.1186/s13071-017-2094-8

**Published:** 2017-03-24

**Authors:** Lenka Minichová, Zuzana Hamšíková, Lenka Mahríková, Mirko Slovák, Elena Kocianová, Mária Kazimírová, Ľudovít Škultéty, Katarína Štefanidesová, Eva Špitalská

**Affiliations:** 1 Institute of Virology, Biomedical Research Center, Slovak Academy of Sciences, Dúbravská cesta 9, 845 05 Bratislava, Slovakia; 2 Institute of Zoology, Slovak Academy of Sciences, Dúbravská cesta 9, 845 06 Bratislava, Slovakia

**Keywords:** *Rickettsia* spp., *Coxiella burnetii*, Rodents, Ticks, Slovakia

## Abstract

**Background:**

Natural foci of tick-borne spotted fever group (SFG) rickettsiae of public health concern have been found in Slovakia, but the role of rodents in their circulation is unclear. Ticks (*Ixodes ricinus*, *Ixodes trianguliceps*, *Dermacentor marginatus*, *Dermacentor reticulatus*, *Haemaphysalis concinna* and *Haemaphysalis inermis*) and tissues of rodents (*Apodemus flavicollis*, *Apodemus sylvaticus*, *Myodes glareolus*, *Microtus arvalis*, *Microtus subterraneus* and *Micromys minutus*) were examined for the presence of SFG rickettsiae and *Coxiella burnetii* by molecular methods. Suburban, natural and rural habitats were monitored to acquire information on the role of ticks and rodents in the agents’ maintenance in various habitat types of Slovakia.

**Results:**

The overall prevalence of rickettsial infection in questing *I. ricinus* and *D. marginatus* was 6.6% and 21.4%, respectively. *Rickettsia helvetica*, *R. monacensis* and non-identified rickettsial species were detected in *I. ricinus*, whereas *R. slovaca* and *R. raoultii* were identified in *D. marginatus. Rickettsia* spp.-infected *I. ricinus* occurred during the whole tick questing period. *Rickettsia helvetica* dominated (80.5%) followed by *R. monacensis* (6.5%). The species were present in all studied habitats. *Rickettsia slovaca* (66.7%) and *R. raoultii* (33.3%) were identified in *D. marginatus* from the rural habitat. *Apodemus flavicollis* was the most infested rodent species with *I. ricinus*, but *My. glareolus* carried the highest proportion of *Rickettsia*-positive *I. ricinus* larvae. Only 0.5% of rodents (*A. flavicollis*) and 5.2% of engorged *I. ricinus* removed from *My. glareolus, A. flavicollis* and *M. arvalis* were *R. helvetica-* and *R. monacensis*-positive. *Coxiella burnetii* was not detected in any of the tested samples. We hypothesize that rodents could play a role as carriers of infected ticks and contribute to the maintenance of rickettsial pathogens in natural foci.

**Conclusions:**

Long-term presence of SFG *Rickettsia* spp. was confirmed in questing ticks from different habitat types of Slovakia. The results suggest a human risk for infection with the pathogenic *R. helvetica*, *R. monacensis*, *R. slovaca* and *R. raoultii*.

**Electronic supplementary material:**

The online version of this article (doi:10.1186/s13071-017-2094-8) contains supplementary material, which is available to authorized users.

## Background

Ticks (Acari: Ixodidae) are vectors of different pathogenic microorganisms with worldwide occurrence. In Slovakia, *Ixodes ricinus* is a common and widely distributed tick species and known vector of *Borrelia burgdorferi* (*sensu lato*), *Anaplasma phagocytophilum*, “*Candidatus* Neoehrlichia mikurensis”, *Rickettsia helvetica*, *Rickettsia monacensis* and *Babesia* spp. [1–5]. *Dermacentor marginatus*, *Dermacentor reticulatus*, *Haemaphysalis punctata*, *Haemaphysalis concinna* and *Haemaphysalis inermis* are other exophilic tick species that are also involved in the circulation of tick-borne microorganisms in natural foci of this country. *Dermacentor marginatus* prefers steppe and forest-steppe habitats and the karst region, whereas the biotopes of *D. reticulatus* are situated in river basins, shrubs and pastureland. *Haemaphysalis* spp. have a focal distribution [6].

Rickettsiae (Rickettsiales: *Rickettsiaceae*) are gram-negative, obligate intracellular bacteria. The ecology of spotted fever group (SFG) rickettsiae has not been clearly elucidated; some circulate in enzootic or epizootic cycles between wild-living vertebrates and arthropod vectors [7]. In Slovakia, *Rickettsia helvetica* is the most prevalent SFG rickettsia in *I. ricinus* ticks, whereas *R. monacensis* occurs less frequently [5, 8, 9]. *Rickettsia slovaca* and *R. raoultii* are usually associated with *Dermacentor* spp. [5, 10]. The role of *Haemaphysalis* spp. as vectors of *Rickettsia* spp. is unknown.

Small rodents are important hosts for immature stages of ixodid ticks and are considered, e.g., as reservoirs of tick-borne encephalitis virus [11], *Borrelia miyamotoi* and “*Ca*. N. mikurensis” [12] in Europe. Species such as *Microtus agrestis*, *Myodes glareolus*, *Apodemus sylvaticus*, *Apodemus flavicolis* as well as insectivores, e.g., *Sorex araneus*, have been found to be susceptible to *R. helvetica* infection [13]. On the other hand, *R. helvetica* did not show any pathogenic effect on Swiss mice, guinea pigs or domestic rabbits in the laboratory [14]. However, following the bite of infected *D. marginatus*, *R. slovaca* and specific antibodies against this bacterium were found in *A. flavicollis* and *My. glareolus* blood [15, 16].

Humans are only occasional hosts for ticks and rarely play a role in the subsequent transmission of bacteria. *Rickettsia helvetica* is the etiological agent of uneruptive tick bite fever in humans which is characterized by fever, headeache, and myalgia. It may also cause myocarditis and meningitis [17, 18]. *Rickettsia raoultii* is probably less pathogenic than *R. slovaca* [19]; they were described as etiological agents of TIBOLA/DEBONEL/SENLAT disease in humans, which is associated with a tick bite, an inoculum eschar, and cervical lymphadenopathies.

*Coxiella burnetii*, the causative agent of Q fever in humans, is an intracellular bacterium. The most common reservoirs are ruminants, primarily cattle, sheep and goats, although some documented outbreaks have been associated with parturient cats or birds. Humans typically acquire an infection from inhaling infected aerosols or dust generated by infected animals or animal products [20]. In Slovakia, *C. burnetii* has been isolated and identified from *I. ricinus*, *D. marginatus*, *D. reticulatus*, *H. concinna* and *H. inermis*, but the role of these ticks in its maintenance is unclear [21, 22].

In the present study, we aimed to reveal the presence of SFG rickettsiae in questing ticks, wild-living rodents and rodent-attached ticks in different habitat types in order to identify potential reservoirs and amplifying hosts. The results will be a basis for future epidemiological studies and risk assessment of human tick-borne rickettsioses in Slovakia.

## Methods

### Sample collection

Questing ticks, rodents, and rodent-attached ticks were collected from three study sites in Slovakia. The locations were described in detail in Kazimírová et al. [23] and Berthová et al. [24]. Two study sites (Bratislava and Fúgelka) are located in the Small Carpathian Mountains (southwestern Slovakia). The mountains are partly densely forested with sessile oak (*Quercus petraea*) and European hornbeam (*Carpinus betulus*) dominating at lower altitudes and European beech (*Fagus sylvatica*) dominating at higher altitudes [23]. The recreational forest city park Železná studnička and the campus of the Slovak Academy of Sciences (SAS) at Bratislava (202–334 m above sea level, asl) represent a suburban habitat with significant anthropogenic impact. The natural habitat at Fúgelka (336–436 m asl) is set in a deciduous forest. The third study site located at Prievidza (central Slovakia; 289 m asl), is set nearby a settlement and represents a forest-steppe rural area with Carpathian oak-hornbeam woods [24].

Questing ticks were collected by dragging a 1 m^2^ white wool blanket over the vegetation in monthly intervals during the peak tick questing activity periods, i.e., April - October 2011–2013 in the suburban and natural habitat, and during March - September 2012–2013 in the rural habitat. In each site three 100 m long transects were selected, the blanket was checked for ticks each 5 m. All collected ticks were stored in 1.5 ml tubes filled with 70% ethanol. The species and developmental stage of ticks were identified using the key by Siuda [25]. The impacts of environmental factors, including food availability for rodents, on the abundance and life history of ticks and population dynamics of rodents have not been analysed in this study.

Live-trapping of small rodents was carried out during April - June and September - October 2012–2014 in the suburban and natural habitats [26], and in May - July, and September - October 2012–2013 in the rural habitat. Sherman live traps baited with oat flakes were placed 5 m apart; 100 traps were set in each capture site and trapping event for a total of 1800 trap nights in each study site. Traps were exposed during two consecutive nights and were checked regularly every morning. Captured rodents were transported to the laboratory, anaesthetized and sacrificed humanely according to current laws of the Slovak Republic. This procedure was approved by the Ministry of Environment of the Slovak Republic, Regional Environmental Office in Bratislava (licence ZPO-594/2012-SAB). The rodents were identified to species, sex and age classes (juvenile, subadult, adult). Each rodent individual was screened for the presence of ticks. Collected ticks were stored in 70% ethanol until identification. Blood samples obtained from *sinus orbitalis* and spleen biopsies were stored in 70% ethanol, skin samples from ear were stored at -80 °C.

### Molecular analysis

Genomic DNA of ticks and rodent tissues were extracted using NucleoSpin Tissue kit (Macherey-Nagel, Düren, Germany) according to manufacturer instruction. Presence of *Rickettsia* spp. and *C. burnetii* DNA in the samples was evaluated by PCR assays targeting 381 bp and 401 bp fragments of the citrate synthase gene *gltA* [27, 28] of rickettsiae and a 493 bp fragment of the outer membrane protein *com1* gene of *C. burnetii* [22]. In each reaction positive (DNA of *R. slovaca* and *R. helvetica*, and *C. burnetii* strain Nine Mile originated from the deposit of the Institute of Virology BMC SAS Bratislava) and negative controls (miliQ water) were applied. The primers and cycling conditions are shown in Additional file 1. PCR amplifications were followed by electrophoresis in 1.0% agarose gel stained with GelRed^TM^ (Biotium, Hayward, California). *Rickettsia*-positive tick samples were screened for the presence of *R. helvetica* using TaqMan PCR assay targeting a 65-bp fragment of the 23S rRNA gene as previously described [29], details are shown in Additional file 1. Alternatively, *Rickettsia*-positive amplicons were purified and analysed by sequencing both DNA strands by Macrogen Inc. (Amsterdam, The Netherlands). In the samples with non-identified rickettsial species fragments of 16S rRNA, *sca4, ompA* and *ompB* genes were amplified [30–33] (details are shown in Additional file 1). The DNA sequences were compared with those available in the GenBank database using the Basic Local Alignment Search Tool (Blast) on http://blast.ncbi.nlm.nih.gov/. The new sequences generated in this study were submitted to GenBank under accession numbers KY073144 for *gltA*, KY081649 for *sca4* and KY628821-KY628825, KY628827 for 16S rRNA genes. A phylogenetic analysis was further performed using MEGA5 software [34]. The phylogenetic analysis of *gltA* gene of the samples was carried out using the Neighbor-Joining method [35]. The evolutionary distances were computed using the Maximum Composite Likelihood method [36] and are in the units of the number of base substitutions per site. The rickettsial genes *gltA* and 16S rRNA were concatenated and subjected to analysis by the Neighbor-Joining method. The evolutionary distances were computed using the p-distance method [34].

All PCR-positive questing *D. marginatus* ticks were subsequently analysed for the presence of *sca4* gene [31] (see primers and cycling conditions in Additional file 1). To distinguish *R. slovaca* from other rickettsial species PCR-RLFP analysis was employed. Amplified *sca4* gene was digested using *HaeIII* restriction endonuclease (digested fragments for *R. slovaca* are 477 bp and 146 bp [37]). After 3 h of incubation at 37 °C, samples were loaded in 1% agarose gel and separated using electrophoresis. Undigested samples were purified and both strands were sequenced by Macrogen Inc. (Amsterdam, The Netherlands).

### Statistical analysis

For rodents, we determined tick infestation prevalence (P, the proportion between the number of rodents with ticks and the number of captured rodents) and intensity of parasitization (I, the number of ticks per infested rodent). Statistical analyses to test the differences in the prevalences of infection with rickettsiae between years and sites were carried out with the Fisher’s exact test using Past version 2.17b software [38]. A *P*-value < 0.05 was considered significant. 95% confidence intervals (CI) were calculated for prevalences. Differences in the prevalence of infection with *Rickettsia* spp. in questing and feeding ticks were analyzed between sexes and habitats applying Fisher’s exact test, supplemented with Mantel-Haenszel common odds ratio estimate (OR) and its 95% confidence interval in cases when two prevalences were compared.

## Results

### Overall rate of *Rickettsia* infections in ticks and rodents

In total, 8593 questing ticks of four species (*I. ricinus*, *D. marginatus*, *H. concinna* and *H. inermis*) were collected during the study, with the dominance of *I. ricinus*. A total of 663 rodents of 6 species (*Apodemus flavicollis*, *Apodemus sylvaticus*, *Myodes glareolus*, *Microtus arvalis*, *Microtus subterraneus*, *Micromys minutus*) were captured. From rodents, in total 1264 ixodid ticks were collected (1208 larvae, 50 nymphs, 6 adults). The dominant species was *I. ricinus* (93.43%; 1132 larvae, 48 nymphs, and 1 female), followed by *H. concinna* (6.17%; 73 larvae and 5 females), *I. trianguliceps* (0.32%; 2 larvae, 2 nymphs) and one larva of *D. reticulatus*. Rodent-attached *I. ricinus* larvae clearly prevailed over nymphs.

Randomly selected 4336 (3983 *I. ricinus*, 182 *D. marginatus*, 166 *H. concinna*, 5 *H. inermis*) questing ticks were individually screened for the presence of *Rickettsia* spp. and *C. burnetii*. DNA of *C. burnetii* was not confirmed in any sample, whereas the presence of rickettsial DNA was confirmed in 301 (6.9%, 95% CI: 6.2–7.7) ticks. Only *I. ricinus* and *D. marginatus* were found to be *Rickettsia*-positive. Infected ticks were found in all habitats (Table 1). The overall prevalence of infection in *D. marginatus* (21.4%, 95% CI: 15.5–27.4) was significantly higher than in *I. ricinus* (6.6%, 95% CI: 5.8–7.3) (*P* < 0.001; OR = 3.8; 95% CI: 2.6–5.5).

**Table 1:** Prevalence^a^ of *Rickettsia* spp*.* in questing ticks in three habitat types in Slovakia in 2011–2013

Habitat/tick stage	2011	2012	2013	Total
*Ixodes ricinus*				
Suburban (total)	45/859 (5.2)^a^	22/312 (7.1)	53/645 (8.2)	120/1,816 (6.6)
Females	10/99 (10.1)	8/49 (16.3)	15/102 (14.7)	33/250 (13.2)
Males	14/109 (12.8)	5/68 (7.4)	4/98 (4.1)	23/275 (8.4)
Nymphs	21/651 (3.2)	9/195 (4.6)	34/445 (7.6)	64/1,291 (5.0)
Natural (total)	83/1,054 (7.9)	22/388 (5.7)	28/398 (7.0)	133/1,840 (7.2)
Females	13/85 (15.3)	4/52 (7.7)	6/61 (9.8)	23/198 (11.6)
Males	11/102 (10.8)	5/66 (7.6)	4/74 (5.4)	20/242 (8.3)
Nymphs	59/867 (6.8)	13/270 (4.8)	18/263 (6.8)	90/1,400 (6.4)
Rural (total)		0/8	9/319 (2.8)	9/327 (2.8)
Females		0/3	0/14	0/17
Males			1/16 (6.3)	1/16 (6.3)
Nymphs		0/1	8/103 (7.8)	8/104 (7.7)
Larvae		0/4	0/186	0/190
Total	128/1,913 (6.7)	44/700 (6.3)	90/1,362 (6.6)	262/3,983 (6.6)
*Dermacentor marginatus*				
Rural (total)		17/147 (11.6)	22/35 (62.9)	39/182 (21.4)
Females		7/73 (9.6)	14/21 (66.7)	21/94 (22.3)
Males		10/74 (13.5)	8/13 (61.5)	18/87 (20.8)
Nymphs			0/1	0/1

^a^No. of *Rickettsia*-positive/no. of tested ticks (percentage of *Rickettsia*-positive ticks %)

The total *Rickettsia* prevalence in questing *I. ricinus* did not differ significantly between the years of study. The prevalence in *I. ricinus* females (12.0%, 95% CI: 9.1–15.0) was significantly higher than in males (8.3%, 95% CI: 5.9–10.6) (*P* = 0.048; OR = 1.5; 95% CI: 1.0–2.3) and was also higher in adults (10.0%, 95% CI: 8.2–11.9) than in nymphs (5.8%, 95% CI: 4.9–6.7) (*P* < 0.001; OR = 1.8; 95% CI: 1.4–2.3). The prevalence in *I. ricinus* in the suburban (6.6%, 95% CI: 5.5–7.8) and natural (7.2%, 95% CI: 6.0–8.4) habitats did not differ significantly (*P* = 0.460; OR = 0.9; 95% CI: 0.7–1.2), but the prevalence of infection was significantly higher in the suburban and natural habitats compared to the rural habitat (2.8%, 95% CI: 1.0–4.5), (6.6 *vs* 2.8%; *P* = 0.009; OR = 2.5; 95% CI: 1.2–5.0 and 7.2 *vs* 2.8%; *P* = 0.003; OR = 2.7; 95% CI: 1.4–5.5).

Altogether 262 *Rickettsia*-positive questing *I. ricinus* samples were analysed by sequencing or by *R. helvetica*-specific real time PCR. *Rickettsia helvetica* was the dominant species (80.5%) present in all tick developmental stages. *Rickettsia monacensis* was identified in 17 (6.5%) ticks of all developmental stages. In 7 (2.7%) ticks, two different unidentified *Rickettsia* spp. were detected (Table 2).

**Table 2:** Proportion^a^ of different *Rickettsia* spp. in questing *Ixodes ricinus* in three habitat types in Slovakia

	*R. helvetica*				*R. monacensis*				*Rickettsia* spp.			
Habitat	Males	Females	Nymphs	Total	Males	Females	Nymphs	Total	Males	Females	Nymphs	Total
Suburban	16/23 (69.6)	28/33 (84.9)	44/64 (68.8)	88/120 (73.3)	3/23 (13.0)	1/33 (3.0)	2/64 (3.1)	6/120 (5.0)	4/23 (17.4)	4/33 (12.1)	18/64 (28.1)	26/120 (21.7)
Natural	17/20 (85.0)	19/23 (82.6)	80/90 (88.9)	116/133 (87.2)	2/20 (10.0)	1/23 (4.4)	8/90 (8.9)	11/133 (8.5)	1/20 (5.0)	3/23 (13.0)	2/90 (2.2)	6/133 (4.5)
Rural			7/9 (77.8)	7/9 (77.8)			0/9	0/9	1/1 (100)		1/8 (12.5)	2/9 (22.2)
Total	33/43 (76.7)	47/56 (83.9)	131/163 (80.4)	211/262 (80.5)	5/43 (11.6)	2/56 (3.6)	10/163 (6.1)	17/262 (6.5)	6/43 (14.0)	7/56 (12.5)	21/163 (12.9)	34/262 (13.0)

^a^No. of *Rickettsia*-positive/no. of tested ticks (percentage of *Rickettsia*-positive ticks %)

A total of 1090 rodent-attached ticks (1024 *I. ricinus*, 61 *H. concinna*, 4 *I. trianguliceps*, 1 *D. reticulatus*) of all stages were screened for the presence of rickettsiae. All *Rickettsia*-positive ticks feeding on rodents were *I. ricinus*, with the highest prevalence in the rural habitat (6.5%, 95% CI: 1.5–11.6), followed by the suburban (6.1%, 95% CI: 4.0–8.1) and the natural habitat (3.7%, 95% CI: 1.9–5.5) (Table 3). However, the total prevalence did not differ significantly between the habitats (6.5 *vs* 6.1%; *P* = 0.860; OR = 1.1; 95% CI: 0.4–2.7; 6.5 *vs* 3.7%; *P* = 0.241; OR = 1.8; 95% CI: 0.7–4.7; and 6.1 *vs* 3.7%; *P* = 0.117; OR = 1.7; 95% CI: 0.9–3.1).

**Table 3:** Prevalence^a^ of *Rickettsia* spp. in rodent-attached ticks from three habitat types in Slovakia

Habitat	*I. ricinus*			*H. concinna*		*I. trianguliceps*		*D. reticulatus*
Year	L	N	A	L	A	L	N	L
Suburban (Total)	29/505 (5.7)	3/21 (14.3)		0/22	0/1	0/2	0/2	
2012	18/213 (8.5%)			0/17				
2013	1/30 (3.3)	3/21 (14.3)			0/1			
2014	10/262 (3.8)			0/5		0/2	0/2	
Natural (Total)	15/400 (3.8)	0/6	0/1	0/34	0/3			0/1
2012	12/169 (7.1)	0/1	0/1	0/16	0/3			
2013	1/5 (20.0)							
2014	2/226 (0.9)	0/5		0/18				0/1
Rural (Total)	6/88 (6.8)	0/3		0/1				
2012	3/43 (7.0)	0/1						
2013	3/45 (6.7)	0/2		0/1				
Total	50/993 (5.0)	3/30 (10.0)	0/1	0/57	0/4	0/2	0/2	0/1

*Abbreviations:* *A* adults, *L* larvae, *N* nymphs

^a^No. of *Rickettsia*-positive/no. of tested ticks (percentage of *Rickettsia*-positive ticks %)

*Ixodes ricinus* infestation prevalence and intensity of parasitization on *A. flavicollis* were higher than on *My. glareolus* (Table 4), but the prevalence of rickettsial infection was higher in ticks collected from *My. glareolus* than from *Apodemus* spp. (Table 5). The highest prevalence of rickettsial infection was determined in ticks collected from *M. arvalis*, but the total number of ticks collected from these rodents was only 28.

**Table 4:** Tick infestation prevalence and intensity of parasitisation of rodents by ticks in three habitat types in Slovakia

Habitat	Rodent species	*I. ricinus*	
		P (%)	I
Suburban	*Apodemus flavicollis*	69.44	3.42
	*Apodemus sylvaticus*	100	7
	*Myodes glareolus*	39.50	1.94
Natural	*Apodemus flavicollis*	64.20	3.12
	*Apodemus sylvaticus*	100	1
	*Myodes glareolus*	20.37	1.55
	*Micromys minutus*	100	2
	*Microtus arvalis*	36.84	2.43
Rural	*Apodemus flavicollis*	48.9	4.18
	*Myodes glareolus*	0	0

*Abbreviations:* *P* tick infestation prevalence, *I* intensity of parasitization

**Table 5:** Presence^a^ of *Rickettsia* spp. in ticks attached to individual rodent species captured in three habitat types in Slovakia

Habitat	*A. flavicollis*	*A. sylvaticus*	*My. glareolus*	*M. arvalis*	*M. minutus*	Total
Suburban						
IRN	3/0/0/21 (14.3)					3/0/0/ 21 (14.3)
IRL	21/1/0/407 (5.4)	0/7	7/0/0/91 (7.7)			28/1/0/505 (5.7)
ITL			0/2			0/2
ITN	0/1		0/1			0/2
HCL	0/13		0/9			0/22
HCA	0/1					0/1
Total	24/1/0/443 (5.6)	0/7	7/0/0/103 (6.8)			31/1/0/553 (5.8)
Natural						
IRN	0/5			0/1		0/6
IRL	7/1/0/348 (2.3)	0/1	2/1/0/34 (8.8)	4/0/0/15 (26.7)	0/2	13/2/0/400 (3.8)
IRA				0/1		0/1
HCL	0/20		0/6	0/8		0/34
HCA				0/3		0/3
DRL	0/1					0/1
Total	7/1/0/374 (2.1)	0/1	2/1/0/40 (7.5)	4/0/0/28 (13.8)	0/2	13/2/0/445 (3.4)
Rural						
IRN	0/3					0/3
IRL	4/1/1/88 (6.8)					4/1/1/88 (6.8)
HCL	0/1					0/1
Total	4/1/1/92 (6.5)					4/1/1/92 (6.5)

*Abbreviations:* *IRN I. ricinus* nymphs, *IRL I. ricinus* larvae, *IRA I. ricinus* adults, *ITL I. trianguliceps* larvae, *ITN I. trianguliceps* nymphs, *HCL H. concinna* larvae, *HCA H. concinna* adults, *DRL D. reticulatus* larvae

^a^No. of *R. helvetica*-positive ticks/No. of *R. monacensis*-positive ticks/No. of *Rickettsia* sp.-positive ticks/No. of tested rodent-attached ticks (percentage of *Rickettsia*-positive ticks %)

A collection of randomly selected 640 (282 from suburban, 266 from natural, 92 from rural habitat) rodent-attached ticks were screened for the presence of *C. burnetii,* with negative results.

Ear biopsies and/or spleen and/or blood samples from rodents were analysed for the presence of rickettsiae and *C. burnetii*. DNA of *Rickettsia* spp. was found in 0.5% (95% CI: 0.1–1.0; 3/663) rodents. All were *A. flavicollis*. None of the rodent samples was found to be PCR-positive for *C. burnetii*.

### Suburban habitat

In the suburban habitat questing *I. ricinus* dominated (for details see [23]). Three hundred rodents were captured (for details on species composition see [26]), and 553 rodent-attached ticks (*I. ricinus*, *H. concinna* and *Ixodes trianguliceps*) were collected (Table 3).

**Fig. 1:**
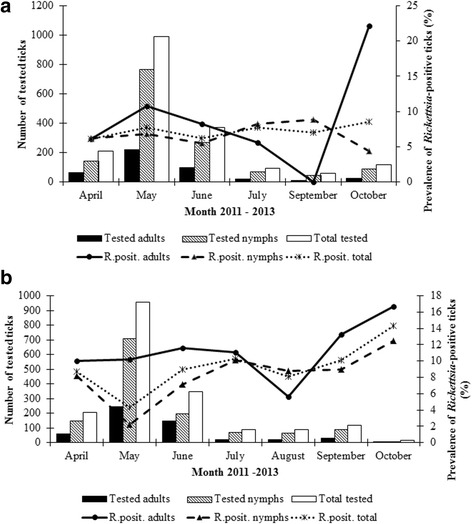
Seasonal distribution of *Rickettsia*-positive questing *I. ricinus* ticks in the suburban (**a**) and natural (**b**) habitats during 2011–2013

The total *Rickettsia* infection rate in questing *I. ricinus* was 6.6% (Table 1). No significant differences between years were found for females, but significant differences in overall prevalence and in prevalence in males and nymphs were revealed between 2011 and 2013 (*P* = 0.021, *P* = 0.025 and *P* = 0.001, respectively). The difference in infection rate in females and males was not significant (13.2 *vs* 8.4%; *P* = 0.075; OR = 1.7; 95% CI: 1.0–2.9), but it was significant between adults and nymphs (11.9 *vs* 5%; *P* < 0.001; OR = 2.3; 95% CI: 1.6–3.3). Analysis of seasonal distribution of *Rickettsia* spp.-infected *I. ricinus* during 2011–2013 showed that infected ticks occurred during the whole questing period (Fig. 1a). The highest total infection rate (10.3%, 95% CI: 3.9–16.7) was observed in July and the lowest one (4.3%, 95% CI: 3.0–5.6) in May. The difference was significant (*P* = 0.014; OR = 2.6; 95% CI = 1.2–2.5). In nymphs, the maximum prevalence was determined in July (10.1%, 95% CI: 3.0–17.3), but in adults it was in September (13.3%, 95% CI: 1.2–25.5). October was not considered in the seasonal analysis because of low numbers of tested ticks.

**Fig. 2:**
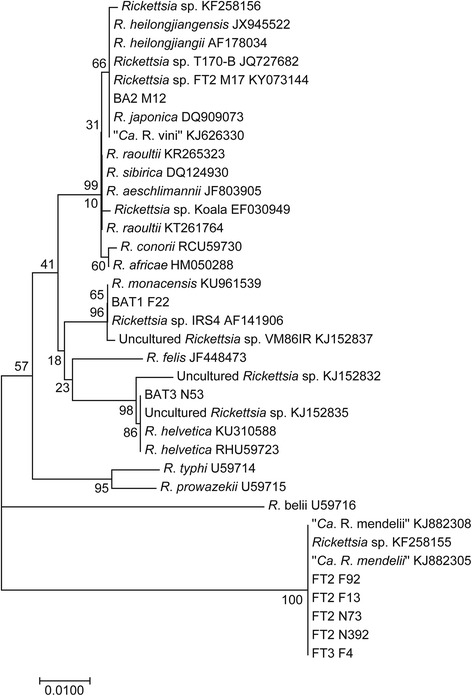
Phylogenetic tree inferred from comparison of the *Rickettsia gltA* partial sequences. GeneBank accession numbers are included

*Rickettsia helvetica* was the dominant rickettsial species. This species was identified in 88 ticks (73.3%) whereas *R. monacensis* was identified in 6 ticks (5.0%) (representative sequences from samples BAT1 F22 and BAT3 N53, Fig. 2). An unidentified *Rickettsia* sp. was found in one male *I. ricinus* (sample BA2 M12) (0.8%). Partial sequencing of the *gltA* gene showed 100% identity with *Rickettsia japonica* (GenBank DQ909073) previously found in patient blood in Thailand [39], and 99% similarity with the *Rickettsia* sp. from an *I. ricinus* male (FT2 M17, GenBank KY073144) from the natural habitat as described below. The quality of the *sca4* gene sequences of sample BA2 M12 from this study was low. DNA of 25 *Rickettsia-*positive ticks, but negative by *R. helvetica*-specific real-time PCR, were not sequenced.

*Rickettsia helvetica* and *R. monacensis* were identified in rodent-attached *I. ricinus* (Table 5) and in ear biopsies from two *A. flavicollis* females, which were not infested by ticks.

### Natural habitat

In the natural habitat, questing *I. ricinus* clearly dominated over *H. concinna* (for details see [23]). Three hundred and six rodent specimens were captured (for details on species composition see [26]) and 445 rodent-attached ticks (*I. ricinus, H. concinna* and *D. reticulatus*) were collected (Table 3).

**Fig. 3:**
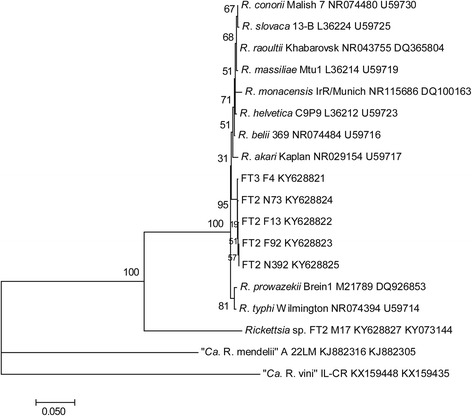
Phylogenetic tree inferred from comparison of concatenated *gltA* and 16S rRNA *Rickettsia* partial sequences. GeneBank accession numbers are included

The total *Rickettsia* infection rate in questing *I. ricinus* was 7.2% (Table 1). Infection rates did not differ significantly between years. The difference between prevalence of infection in female and male ticks was not significant (11.6 *vs* 8.3%; *P* = 0.240; OR = 1.4; 95% CI: 0.8–2.7), but it was significant between adults and nymphs (10.8 *vs* 6.4%; *P* = 0.019; OR = 1.6; 95% CI: 1.1–2.3). *Rickettsia*-infected *I. ricinus* were present during the whole investigation period (Fig. 1b). The highest total prevalence was observed in October (8.5%, 95% CI: 3.4–13.5) and the lowest in June (6.2%, 95% CI: 3.7–8.7), but without significant difference (*P* = 0.393; OR = 1.4; 95% CI: 0.6–3.0). In nymphs, the maximum prevalence was observed in July (8.2%, 95% CI: 1.9–14.5), and in adults in May (10.8%, 95% CI: 6.7–14.9); October was not included in the seasonal analysis for adults because of the low number of tested specimens (27). Out of the *Rickettsia* spp.-positive *I. ricinus* ticks, the presence of *R. helvetica* was identified in 87.2%, *R. monacensis* in 8.3% and two different unidentified *Rickettsia* spp. in 3.8% (*n* = 5) and 0.8% (*n* = 1) specimens. The unidentified *Rickettsia* spp. were found in three *I. ricinus* females (DNA isolates marked as FT2 F92, FT2 F13, FT3 F4) and two nymphs (DNA isolates FT2 N73, FT2 N392). PCR products of the expected size were obtained with primers targeting *gltA,* 16S rRNA and *ompB genes*, but no product using *sca4* and *ompA* primers was obtained. Partial sequencing of the *gltA* gene of the unidentified *Rickettsia* spp. showed 100% identity with “*Candidatus* Rickettsia mendelii” (GenBank KJ882305) identified in *I. ricinus* captured from *Luscinia megarhynchos* in the Czech Republic and *Rickettsia* sp. 450IRF BA (GenBank KF258155) identified in an *I. ricinus* female collected in Bratislava [8]. By BLAST analysis, 16S rRNA partial sequence of the *I. ricinus* rickettsial samples was most similar (98–99%) to the corresponding sequence of *R. belii* (GenBank NR074484) and *R. prowazekii* (GenBank NR044656). The quality of the *ompB* gene fragments of the samples was low. Another unidentified *Rickettsia* sp. was found in an *I. ricinus* male (DNA isolate FT2 M17, GenBank KY073144) (Fig. 2). Partial sequencing of the *gltA* gene showed 99% similarity with unidentified *Rickettsia* sp. (GenBank KF258156) previously found in an *I. ricinus* male in Bratislava [8], *R. raoultii* (GenBank KT261764) found in *D. marginatus* in China, *R. japonica* (GenBank DQ909073) identified from a human in Thailand [39] and with *R. heilongjiangii* (GenBank AF178034) [40]. Figure 2 shows a phylogenetic tree constructed on the basis of the *gltA* sequences. Figure 3 presents molecular phylogenetic analysis on the basis of concatenated *gltA* and 16S rRNA genes. Partial sequencing of the *sca4* gene from the FT2 M17 DNA isolate (GenBank KY081649) showed 99% similarity with *Rickettsia* sp. AUS118 (GenBank KF666473) form *Argas lagenoplastis,* with the endosymbiont *Rickettsia peacockii* str. Rustic (GenBank CP001227), and *Rickettsia* sp. VF113DR from a *D. reticulatus* female collected in Vojka nad Dunajom (GenBank KJ152839) [5].

*Rickettsia helvetica* and *R. monacensis* were identified in rodent-attached *I. ricinus* ticks (Table 5). *Rickettsia helvetica* was found in spleen from one *A. flavicollis* female, which was not infested by ticks.

### Rural habitat

The highest diversity of tick species was recorded in this habitat: *I. ricinus*, *D. marginatus*, *H. concinna* and *H. inermis. Ixodes ricinus* dominated (*n* = 327), followed by *D. marginatus* (*n* = 182), *H. concinna* (*n* = 95: 24 nymphs and 71 larvae) and *H. inermis* (5 females)*.* Only *A. flavicollis* (*n* = 45: 30 females, 15 males) and *My. glareolus* (*n* = 12: 6 females, 6 males) were caught. Fourty *A. flavicollis* and 12 *My. glareolus* were caught in 2012 and 5 *A. flavicollis* in 2013. In total, 92 rodent-attached *I. ricinus* and *H. concinna* ticks were analysed (Table 3).

The total *Rickettsia* spp. infection rate in questing *I. ricinus* was 2.8%; of these, 77.8% were *R. helvetica* (Tables 1 and 2)*.* Two *Rickettsia-*positive ticks, but negative by *R. helvetica*-specific real-time PCR, were not sequenced. *Rickettsia*-infected *I. ricinus* (9.5%; 95% CI: 3.6–15.4) were found only in June.

*Rickettsia*-positive questing *D. marginatus* (total prevalence 21.4%; 95% CI: 15.5–27.4) (Table 1) were found in March, April 2012 and September 2013. The prevalence in *D. marginatus* females and males did not differ significantly (22.3 *vs* 20.8%; *P* = 0.787; OR = 1.1; 95% CI: 0.5–2.2), but it differed significantly between 2012 and 2013. Altogether, 66.7% (14 females, 12 males) of *D. marginatus* were infected with *R. slovaca* and 33.3% (7 females, 6 males) with *R. raoultii*.

*Apodemus flavicollis* was the only tick-infested rodent species (Table 4)*. Rickettsia helvetica*, *R. monacensis* and *Rickettsia* sp. (*R. helvetica*-negative, not analysed by sequencing) were identified in rodent-attached *I. ricinus* ticks (Tables 3 and 5).

## Discussion

In the present study, we screened questing ticks, rodent-attached ticks and rodents for rickettsial and *C. burnetii* infections in three different habitat types in Slovakia. Rickettsial infection was detected in questing and rodent-attached ticks, and in skin and spleen samples derived from *A. flavicollis. Rickettsia helvetica* and *R. monacensis* were identified in questing and rodent-attached *I. ricinus* ticks and in rodent samples. Non-identified *Rickettsia* spp. were found in seven *I. ricinus* ticks, but their subsequent analysis failed because of low quality sequenced data or lack of DNA. *Rickettsia slovaca* and *R. raoultii* were identified only in questing *D. marginatus* ticks. No evidence of *C. burnetii* infections was found in ticks or rodents in any of the studied habitats.

*Ixodes ricinus* is the most common tick in Slovakia and also dominated in all study sites. The highest diversity of tick species was observed in the rural habitat, where in addition to the dominant *I. ricinus*, *D. marginatus*, *H. concinna* and *H. inermis* were found*.* Sympatric occurrence of different tick species was previously recorded in a few other sites of southwestern Slovakia [5, 41]. Similarly as in our study, *I. ricinus* was the dominant species in Martinský forest [41], whereas *D. reticulatus* dominated in the Vojka nad Dunajom flood-plain forest [5]. Thus, the differences in tick abundance and species spectrum in the studied habitats probably depend on specific habitat characteristics, including vertebrate host diversity.

The presence of rickettsial DNA was confirmed in 6.9% of questing ticks. The overall prevalence of rickettsiae was 21.4 and 6.6% in *D. marginatus* and *I. ricinus*, respectively. These findings are in accordance with the results of previous studies from urban, suburban, natural and agricultural sites of Slovakia [5, 8–10].

During 2011–2013, the annual prevalence of rickettsiae in *I. ricinus* ranged from 5.2 to 8.2% in the suburban habitat and from 5.7 to 7.9% in the natural habitat. Similar data (from 6.7 to 8.7%) were reported from an agricultural site of eastern Slovakia [9] and in the Netherlands where the annual prevalence of *R. helvetica* was practically constant during the years 2000–2008 [42].

Analysis of the seasonal distribution of *Rickettsia*-infected *I. ricinus* showed that infected ticks were present during the whole tick questing period in the suburban and the natural habitat, similarly as in an agricultural site of Slovakia [9]. Seasonal/monthly variation in prevalence of *Rickettsia*-infected *I. ricinus* was observed not only in the suburban and natural habitat included in this study, but also in the agricultural site in eastern Slovakia [9], in Germany [43–46], Denmark [47] and the Netherlands [42]. The observed seasonal differences in the prevalence of rickettsial infections in questing ticks could be due to many factors such as tick phenology, sex ratio, habitat type, microclimate, presence and diversity of tick host species.

The initial infection of ticks with rickettsiae occurs when uninfected ticks feed on rickettsemic hosts. The probability to be infected by rickettsiae rises depending on the quantity of blood the tick ingests and the duration of its attachment. The exact process of natural infection of ticks is unrevealed for the majority of tick-borne rickettsiae, but it was observed that all subsequent life stages of the tick would be infected by transstadial transmission [7] and the ticks might acquire rickettsial infection even by co-feeding [48].

Although rodents have been confirmed as reservoirs and amplifying hosts for a number of tick-borne microorganisms [11, 12], their reservoir role has not been resolved in the case of rickettsiae. In our study, we found only 0.5% *R. helvetica*-infected *A. flavicollis,* whereas 4.9% of ticks attached to *A. flavicollis*, *My. glareolus* and *M. arvalis* were *Rickettsia-*positive. Our results support previous findings from Slovakia where rickettsial infection was confirmed only in a low proportion of wild-living rodents [49], or no infection was found [50]. Similarly, in a sylvatic habitat of west-central Poland, rickettsial DNA was detected in 8% of rodent-attached *I. ricinus* nymphs and in 10.7% of larvae, but none of the tested *A. flavicollis* and *My. glareolus* blood samples were positive [51]. However, *R. helvetica* has frequently been detected in ticks feeding on non-rickettsiemic rodents. For example, in central Germany, SFG rickettsiae (mainly *R. helvetica*) were found in 1.8% of *I. ricinus* collected from *A. flavicollis* and *My. glareolus* [52], but in southern Germany ticks feeding on *M. arvalis* were *C. burnetii-* and *Rickettsia* spp.-negative [53]. In Switzerland, *Rickettsia* spp. were detected in 6.8% of immature *I. ricinus* ticks attached to *A. flavicollis* and *My. glareolus*, whereby 20.7% of larvae were infected with *R. helvetica*, but none of the xenodiagnostic ticks fed on rodents were found infected, suggesting that rodents are not amplifying hosts for SFG rickettsieae [12]. In contrast, as much as 29% blood samples obtained from *A. sylvaticus* and *My. glareolus* in the Netherlands were found to be infected with *R. helvetica*, 14% with *Rickettsia* spp. and 1.4% with *Rickettsia conorii* [42]. In south-eastern Germany, rickettsial DNA was found in 7.6% of ear skin samples from rodents [54] and in Hungary, *R. helvetica* was found in 1.9% of *Mus musculus* and in 20% of *Leptopsylla segnis* fleas [55]. During field studies in Italy, 22% of ear biopsies from *Apodemus* spp., but none from *My. glareolus* were *R. slovaca*-infected and the prevalence of *R. slovaca* in host-seeking *D. marginatus* larvae was 42% [56], suggesting that *Apodemus* spp. might play a role as amplifiers of the *R. slovaca* infection. However, based on the published data on rickettsial infections in wild-living rodents it is difficult to define their exact role in the maintenance of *Rickettsia* spp. in natural foci. We can only speculate that rickettsial infections of rodent-attached ticks are the result of either a vertical route of infection (transovarial and/or transstadial transmission) in ticks or a very short rickettsiaemia in rodents. Nevertheless, the presence of pathogenic *R. helvetica*, *R. monacensis*, *R. slovaca* and *R. raoultii* in ticks indicate their potential epidemiological and epizootological significance in the studied sites.

Although *C. burnetii* was previously detected in an *A. flavicollis* specimen from Central Slovakia [49], in this study, the presence of *C. burnetii* was not confirmed in any of the studied samples from ticks or rodents. Similarly, no evidence of *C. burnetii* infections could be found in ticks or rodents in southern Germany [53], suggesting that rodents do not play any essential role in the epidemiology of Q fever. On the other hand, in central Italy *C. burnetii* was detected in blood samples of *Apodemus* spp. and in rodent-attached *I. ricinus* and *Ixodes acuminatus* ticks [57]. These data may suggest that *Apodemus* spp. play a role in the maintenance of Q fever in the focus in the studied area. Moreover, wild-living rodents in Heilongjiang Province (border of China and Russia) [58] and in a Canadian Natural Environment Park [59] were found to be naturally infected with *C. burnetii* and thus are suggested as reservoirs of this patogen.

The presence of different pathogenic tick-borne *Rickettsia* spp. such as *R. helvetica*, *R. monacensis*, *R. raoultii* and *R. slovaca* was confirmed in suburban, natural and rural habitats of Slovakia, which are used by humans for relax (cycling, dog walking, jogging), or hunting. Thus, our data about the presence of various pathogenic rickettsiae in ticks and rodents indicate their potential epidemiological as well as epizootological significance in Slovakia. Therefore, humans visiting the study sites alone or with their pets should be aware of the possible risk of tick infestation and contracting tick-borne rickettsiae. The knowledge and early detection of infections in ticks and animal sources can prevent the transmission of pathogenic rickettsiae to humans.

## Conclusions

The presence of *Rickettsia* spp. was confirmed in questing and rodent-attached *I. ricinus* and *D. marginatus* ticks in south-western and central Slovakia. The infection rates and presence of *Rickettsia* species depended on the habitat type, year, season, tick species and developmental stage. The presence of the pathogenic *R. helvetica*, *R. monacensis*, *R. slovaca* and *R. raoultii* in ticks indicate their potential epidemiological and epizootological significance and the risk of acquiring human rickettsial infections in different habitat types in Slovakia. Wild-living rodents could play a role as carriers of infected ticks and contribute to maintenance of rickettsial pathogens in natural foci.

## Additional file

Additional file 1: PCR protocols for the detection of *Rickettsia* spp. and *Coxiella burnetii. (DOCX 21 kb)*

## Abbreviations

CI: 95% confidence interval
OR: Mantel-Haenszel common odds ratio estimate
SFG: Spotted fever group
