# Supplementary material for: Molecular evidence of Rickettsia spp. in ixodid ticks and rodents in suburban, natural and rural habitats in Slovakia
Source: Parasit Vectors. 2017 Mar 24;10:158. doi: 10.1186/s13071-017-2094-8 (PMC5366151; doi:10.1186/s13071-017-2094-8)
Supplement: Additional file 1: — PCR protocols for the detection of Rickettsia spp. and Coxiella burnetii. (DOCX 21 kb) [file 13071_2017_2094_MOESM1_ESM.docx]

**Additional file 1**

**PCR protocols for the detection of *Rickettsia* spp. and *Coxiella burnetii***

**1. PCR amplification to detect *Rickettsia* spp. based on the *gltA* gene**

*Rickettsia* genus-specific RpCS877F (5´-GGGGACCTGCTCACGGCGG-3´) and RpCS1258R (5´-ATTGCAAAAAGTACAGTGAACA-3´) primers were used to amplify a 381 bp fragment. PCR reactions were carried out in a volume of 20 μl containing 4 μl of ticks genomic DNA (cca 50 ng/ μl) and 10 μl of Master mix (DyNAzyme^TM^ PCR Master Mix, Finnzymes, Finland), 1 μl of each primer (final concentration 0.5 μM) and 5 μl of nuclease free water. Amplification was performed on thermocycler PTC-119 200 Peltier Thermal Cycler (MJ Research, Canada). The thermal cycle reaction consisted of an initial 2 min denaturation at 95°C, followed by 35 cycles at 95°C for 20 s, 53°C for 30 s, and 60°C for 2 min, with a final extension at 72°C for 10 min.

Another *Rickettsia* genus-specific CS78 (5´-GCAAGTATCGGTGAGGATGTA-3´) and CS323 (5´-GCTTCCTTAAAATTCAATAAATC-3´) primers were used to amplify an additional 401 bp long fragment of *gltA* gene. PCR reactions were carried out in a volume of 10 μl containing 1 μl of ticks genomic DNA (cca 50 ng/ μl) and 5 μl of Master mix (DyNAzyme^TM^ II PCR Master Mix (Finnzymes), 0,5 μl of each primer (final concentration 0.5 μM) and 3 μl of nuclease free water. Amplification was performed on thermocycler PTC-119 200 Peltier Thermal Cycler (MJ Research, Canada). The thermal cycle reaction consisted of an initial 2 min denaturation at 95°C, followed by 35 cycles at 95°C for 20 s, 52°C for 30 s, and 72°C for 30 s, with a final extension at 72°C for 7 min.

**2. PCR amplification to detect *Rickettsia* spp. based on the *sca4* gene**

*Rickettsia* genus-specific D767f (5´-CGATGGTAGCATTAAAAGCT-3´) and D1390r (5´-CTTGCTTTTCAGCAATATCAC-3´) primers were used to amplify a 623 bp fragment. PCR reactions were carried out in a volume of 20 μl containing 4 μl of ticks genomic DNA (cca 50 ng/ μl) and 10 μl of Master mix (SuperHot PCR Master Mix, Bioron, Germany), 1 μl of each primer (10 μM), 1 μl of 100mM MgCl_2_ and 3 μl of nuclease free water. Amplification was performed on thermocycler PTC-119 200 Peltier Thermal Cycler (MJ Research, Canada). The thermal cycle reaction consisted of an initial 2 min denaturation at 95°C, followed by 35 cycles at 95°C for 20 s, 50°C for 30 s, and 72°C for 30 s, with a final extension at 72°C for 10 min.

**3. PCR amplification to detect *Rickettsia* spp. based on the 16S rRNA gene**

Primers 800f and rP2 were used to amplify rickettsial fragment of 16S rRNA. PCR reactions were carried out in a volume of 20 μl containing 4 μl of ticks genomic DNA (cca 50 ng/ μl) and 10 μl of Master mix (SuperHot PCR Master Mix, Bioron, Germany), 1 μl of each primer (10 μM), 1 μl of 100mM MgCl_2_ and 3 μl of nuclease free water. Amplification was performed on TPersonal Tthermocycler (Biometra, Germany). The thermal cycle reaction consisted of an initial 2 min denaturation at 95°C, followed by 40 cycles at 95°C for 15 s, 42°C for 30 s, and 72°C for 30 s, with a final extension at 72°C for 7 min.

**4. PCR amplification to detect *Rickettsia* spp. based on the *ompA* gene**

Spotted fever group specific primers RR190.70F (5´-ATGGCGAATATTTCTCCAAAAA-3´) and RR190.701R (5´-GTTCCGTTAATGGCAGCATCT-3´) were used to amplify a 632 bp fragment. PCR reactions were carried out in a volume of 20 μl containing 4 μl of ticks genomic DNA (cca 50 ng/ μl) and 10 μl of Master mix (SuperHot PCR Master Mix, Bioron, Germany), 1 μl of each primer (10 μM), 1 μl of 100mM MgCl_2_ and 3 μl of nuclease free water. Amplification was performed on TPersonal Thermocycler (Biometra, Germany). The thermal cycle reaction consisted of an initial 5 min denaturation at 95°C, followed by 35 cycles at 95°C for 20 s, 54°C for 30 s, and 72°C for 30 s, with a final extension at 72°C for 10 min.

**5. PCR amplification to detect *Rickettsia* spp. based on the *ompB* gene**

For detection of a part of *ompB* gene of rickettsiae PCR using primers rompBOF (5´-GTAACCGGAAGTAATCGTTTCGTAA-3´) and rompBOR (5´-GCTTTATAACCAGCTAAACCACC-3´) amplifying 511bp long amplicon were employed. PCR reactions (in total volume of 10 μl) were performed on thermocycler Labcycler (SensoQuest, Germany) containing 5 μl 2x Master mix (DreamTaq Green PCR Master Mix 2x, Thermo Fisher Scientific, USA), 1 μl of each primer (10 μM), 2 μl of nuclease free water and 1 μl of template DNA. The thermal cycle reaction consisted of an initial 13 min denaturation at 95°C, followed by 35 cycles at 95°C for 30 s, 55°C for 30 s, and 72°C for 2 min and ended with a final extension at 72°C for 10 min.

**6. PCR amplification to detect *Coxiella burnetii* based on the *com1* gene**

*C. burnetii*-specific CBCOS (5´-GCTGTTTCTGCCGAACGTAT-3´) and CBCOE (5´-AGACAACGCGGAGGTTTTTA-3´) primers were used to amplify a 493 bp fragment. PCR reactions were carried out in a volume of 10 μl containing 1.5 μl of ticks genomic DNA (cca 50 ng/ μl) and 0.1 μl of polymerase, 1 μl Buffer B1 (HOT Fire Pol^®^ DNA Polymerase, Solis BioDyne, Estonia), 0.5 μl of each primer (10 μM), 0.6 μl of MgCl_2_, 0.1 μl of dNTP and 5.7 μl of nuclease free water. Amplification was performed on thermocycler Labcycler (SensoQuest, Germany). The thermal cycle reaction consisted of an initial 15 min denaturation at 95°C, followed by 35 cycles at 95°C for 40 s, 56°C for 45 s, and 72°C for 40 s, with a final extension at 72°C for 10 min.

**7. TaqMan PCR assay to detect *Rickettsia helvetica* based on the 23S rRNA gene**

The primers and probe used were Rickhelv.147f (5´-TTTGAAGGAGACACGGAACACA-3´) Rickhelv.211r (5´-TCCGGTACTCAAATCCTCACGTA-3´), and Rickhelv.170p (5´-FAM-AACCGTAGCGTACACTTA-MGBNFQ-3´). The real-time PCR mixtures contained 10 μl of the 2 x Master mix (DyNAmo^TM^ Probe qPCR, Finnzymes, Finnland), final concentrations of 100 nM of each primer and 100 nM of the probe, and 4 μl of the template in a total volume of 20 μl. The *R. helvetica*-specific real-time PCR assay was performed using an Bio-Rad CFX96^TM^ Real-Time System, with an initial step of 50°C for 2 min and a denaturation step of 95°C for 15 min, followed by 45 cycles of 95°C for 15 s and 60°C for 1 min.
